# A Possible Link between Infection with Burkholderia Bacteria and Systemic Lupus Erythematosus Based on Epitope Mimicry

**DOI:** 10.1155/2008/683489

**Published:** 2008-08-03

**Authors:** Wei Zhang, Morris Reichlin

**Affiliations:** ^1^Arthritis and Immunology Program, Oklahoma Medical Research Foundation, Oklahoma City, OK 73104, USA; ^2^Department of Medicine, Oklahoma University Health Sciences Center, Oklahoma City, OK 73104, USA

## Abstract

We previously demonstrated that purified polyclonal and monoclonal anti-dsDNA antibodies bind a 15-mer peptide ASPVTARVLWKASHV in ELISA and Dot blot. This 15-mer peptide partial sequence ARVLWKASH shares similarity with burkholderia bacterial cytochrome B 561 partial sequence ARVLWRATH. In this study, we show that purified anti-dsDNA antibodies react with burkholderia fungorum bacterial cell lysates in Western blot. We used anti-dsDNA antibodies to make an anti-dsDNA antibodies affinity column and used this column to purify the burkholderia fungorum bacterial protein. Purified anti-dsDNA antibodies bind specifically to purified bacterial antigen and purified bacterial antigen blocked the anti-dsDNA antibodies binding to dsDNA antigen. Sera with anti-dsDNA antibodies bind specifically to purified bacterial antigen. We obtained protein partial sequence of RAGTDEGFG which is shared with burkholderia bacterial transcription regulator protein sequence. Sera with anti-dsDNA antibodies bind to RAGTDEGFG peptide better than control groups. These data support our hypothesis that the origin of anti-dsDNA antibodies in SLE may be associated with burkholderia bacterial infection.

## 1. INTRODUCTION

Systemic lupus erythematosus (SLE) is an autoimmune disease that affects multiple organ systems. Although the etiology of SLE, and of autoimmunity in general remain unknown, considerable evidence has been accumulated on the pathophysiologic mechanisms, which lead to the failure of distinction between self and nonself and the production of autoantibodies. Genetic, hormonal, and environmental factors have been attributed roles in the etiology of autoimmunity.

Antinuclear antibodies are a hallmark of SLE, whereas anti-dsDNA antibodies are a very specific marker for this disease. High-affinity anti-dsDNA antibodies correlate with disease activity, especially with renal involvement. Over the past several years, it has been clearly demonstrated that anti-dsDNA antibodies have pathogenic potential. Clinical data demonstrate that anti-dsDNA antibody titers correlate with disease activity in a significant number of patients with lupus nephritis, and glomerular eluates from patients with active lupus nephritis contain anti-dsDNA antibodies [1, 2].

The mechanisms responsible for the production of anti-dsDNA antibodies are not understood. The search for crossreactive antigens continues, as these antigens may yield clues to the origin and pathogenesis of anti-dsDNA antibodies.

A major goal of understanding of the structure, origin, and pathogenicity of anti-dsDNA antibodies is to develop novel targeted treatments for SLE.

In previous study, we used a patient anti-dsDNA antibody to select phage clones and four positive clones were found [3]. Those four clones sequence are clone B (ASPVTARVLWKASHV), clone C (VSSLVLLSHGGPHSS), clone D (IMVLCPLWLGTTS), and clone E (AVAHVTSRRVPRWSAA). We demonstrated that purified polyclonal anti-dsDNA antibodies and a monoclonal anti-dsDNA antibody specifically bind a 15-mer clone B peptide ASPVTARVLWKASHV. This chemically synthesized peptide could be recognized by anti-dsDNA antibodies in ELISA and Dot blot [3].

The sequences of the four clones were loaded into the National center for Biotechnology Information (NCBI) protein database to search for similarity to some protein sequence. It is of interest to find that this 15-mer clone B peptide partial sequence ARVLWKASH shares similarity with burkholderia bacterial cytochrome B 561 partial sequence ARVLWRATH (including Burkholderia fungorum, Burkholderia dolosa, Burkholderia cenocepacia, Burkholderia mallei, Burkholderia pseudomallei, Burkholderia thailandensis and Burkholderia cenocepaci). The clone C partial sequence LVLLSHGGPH shares sequence with burkholderia bacterial transcriptional regulator partial sequence.

In this study, we have examined anti-dsDNA antibodies from SLE patient's sera to see whether they can react with Burkholderia bacterial protein. We have purified and isolated bacterial protein and sequenced these proteins. Synthetic peptides have been prepared to confirm that anti-dsDNA antibodies can bind these antigens in vitro. A possible link between an immune response to burkholderia and anti-dsDNA antibody production in lupus patients has been investigated.

## 2. MATERIALS AND METHODS

### 2.1. Patient data

Human sera used in this study were collected from SLE patients and CFII from Sigma (St. Louis, Mo, USA) was used as control antibody. All 30 SLE patients satisfied American College of Rheumatology revised criteria for the classification of SLE [4]. SLE sera were assayed for the quantity of anti-dsDNA by ELISA and Crithidia luciliae anti-dsDNA antibody test kit from HELIX diagnostic (Madison, WI). All 30 SLE patients have anti-dsDNA antibodies.

### 2.2. Preparation of IgG anti-dsDNA antibody and Biopanning of phage display random peptide libraries by anti-dsDNA antibodies

The IgG fraction from anti-dsDNA positive sera was isolated by chromatography on DE-52 and protein A-sepharose (Sigma) [3]. Purified IgG from either DE-52 or protein A was passed through a dsDNA affinity column (Sigma). The column was washed with Tris buffered saline (PH 7.4), and bound protein was eluted with 3 M MgCl_2_. The purified antibodies were assayed for quantity of anti-dsDNA by ELISA and Crithidia luciliae anti-dsDNA antibody test kit (HELIX diagnostic).

A 15-mer peptide library display on gene VIII product of fd phage [5] was used in the study. This library was generously provided by Dr. G. P. Smith (University of Missouri, Columbia, Mo, USA). The basic methods of screening were adapted from the previous reports [6, 7]. Three rounds of screening were performed with the biotinylated anti-dsDNA antibodies. For the first round of selection, 10 ug of biotinylated anti-dsDNA antibodies in 400 *μ*L of 0.5 *χ*TBST (25 mM Tris, 75 mM NaCl and 0.05% Tween 20, pH 7.5) containing 1mg/mL BSA were immobilized onto a streptavidin-coated petri dish by incubation for 2 hours at room temperature. The phage suspension library was added and incubated out 4 hours at room temperature. After the removal of unbound phage by washing 10 times with 0.5 *χ*TBST, the bound phage was recovered using elution buffer (0.1 N HCl-glycine, pH 2.2,). The eluates were immediately neutralized using 2 M Tris. Eluted phage was amplified in Escherichia coli K91 Kan and used as input in the subsequent rounds of selection. After three rounds of selection, phage clones harvested from the third round output were randomly selected and propagated in 1.5 mL of terrific broth [1.2% (w/v) bacto-tryptone, 2.4% (w/v) yeast extract, 0.4% (v/v) glycerol, 17 mM KH_2_PO_4_ and 72 mM K_2_HPO_4_] supplemented with 20 ug/mL tetracycline at 37°C for 20 hours. The cultures were clarified at 2500 g for 10 minutes. The supernatants were recovered for the selection of anti-dsDNA antibody binding clones and subsequent DNA sequencing.

## 3. WESTERN BLOT

Burkholderia fungorum bacterial cells were purchased from ATCC. These bacteria in BBL trypticase soy broth (Becton, Dickinson and Company, Sparks, Md, USA) were cultured overnight at 37°C [8]. The bacterial cell lysates were applied to 12.5% SDS-PAGE gel. After electrophoretic separation, the proteins were transferred to a nitrocellulose membrane. The nitrocellulose membrane was used for screening antibodies reactive with Burkholderia fungorum bacterial proteins.

### 3.1. Preparation of IgG anti-dsDNA antibody column and purification of proteins from Burkholderia fungorum bacterial cell lysates

A purified IgG anti-dsDNA antibody preparation which bound to Burkholderia fungorum bacterial protein was used to prepare an anti-dsDNA antibody affinity column. Purified anti-dsDNA antibodies were coupled to CNBr-activated sepharose 4B [9]. The Burkholderia fungorum bacterial lysates were passed through an anti-dsDNA antibody affinity column. The column was washed with Tris buffered saline (PH 7.4), and bound protein was eluted with 3M MgCl_2_.

### 3.2. Purified human IgG anti-dsDNA antibodies binding to purified burkholderia fungorum bacterial antigen

IMMULON plates from Thermo Scientific (Milford, MA, USA) were used to detect the binding of 5 affinity purified human IgG anti-dsDNA antibodies isolated from SLE patients sera. In addition, one human IgG anti-dsDNA monoclonal antibody, and normal IgG as control were assessed for binding to the purified burkholderia fungorum bacterial antigen. The ELISA plate was coated with purified bacterial antigen (50 ug/mL) and purified anti-dsDNA Ab (1 ug/100 uL) was applied to the plate. Binding of IgG was detected using goat antihuman IgG Fc fragment specific alkaline.

### 3.3. Inhibition of anti-dsDNA binding to dsDNA antigen by the purified bacterial antigen

Reaction mixtures of anti-dsDNA antibody at the same concentration and the purified bacterial antigen at varying concentrations were prepared. The ELISA plate was coated with dsDNA (50 ug/mL). After the purified bacterial antigen was incubated for two hours with anti-dsDNA antibodies at RT, the reaction mixture was transferred to dsDNA-coated wells. The amount of human antibodies bound to the plate was determined by using an antihuman IgG Fc specific alkaline phosphatase conjugate (Sigma). Following another washing, the substrate was added to the plate for color development.

### 3.4. Serum binding to purified bacterial antigen

IMMULON plates were used to detect three groups of sera; 30 SLE patients' sera, secondly 60 control sera that include 15 rheumatoid arthritis sera, 15 polymyositis dermatomyositis sera, 15 sclerodema sera, 15 anti-Ro/anti-La positive sera, and thirdly 30 normal sera. The plates were coated with purified bacterial antigen (50 ug/mL) and incubated at 4°C overnight. The wells were washed and treated with blocking solution. Sera diluted 1/100 with PBS were added to the peptide-coated wells and incubated for 2 hours at room temperature. After washing, binding of IgG was detected using goat antihuman IgG Fc fragment specific alkaline phosphatase conjugate (Sigma).

## 4. PROTEIN SEQUENCING

The eluate from the anti-dsDNA antibody column was applied to 12.5% SDS-PAGE gel and then electrotransferred to PVDF membranes [10]. Proteins bound to PVDF were stained by Coomassie Blue R-250. The stained protein band was excised with a clean razor blade. The protein sequence was determined by Oklahoma University Health Center Molecular Biology-Proteomics Facility.

### 4.1. Peptide synthesis

A protein partial sequence of RAGTDEGFG from the bacterial protein band in PVDF was obtained. The RAGTDEGFG sequence is shared with burkholderia bacterial transcription regulator protein sequence.The peptide RAGTDEGFG and a scrambled form as control peptide FGAGTRDGE were synthesized by the Molecular Biology Resource Facility, University of Oklahoma Health Sciences Center. The purity of the peptides was = >90% by reverse phase HPLC and the compositions were confirmed by amino acid analysis.

### 4.2. Purified human IgG anti-dsDNA antibodies binding to synthetic peptide

IMMULON plates were used to detect the binding of 5 affinity purified human IgG anti-dsDNA antibodies isolated from SLE patients sera. In addition, one human IgG anti-dsDNA monoclonal antibody, and normal IgG as control were assessed for binding to the synthetic peptide (RAGTDEGFG) and control peptide (FGAGTRDGE). The ELISA plate was coated with synthetic peptide (50 ug/mL) and purified anti-dsDNA antibody (1 ug/100 uL) was applied to the plate. Binding of IgG was detected using goat antihuman IgG Fc fragment specific alkaline phosphatase conjugate (Sigma).

### 4.3. Inhibition

Inhibition of anti-dsDNA binding to dsDNA antigen by the synthetic peptide.

Reaction mixtures of anti-dsDNA antibody at the same concentration and the synthetic peptide at varying concentrations were prepared. The ELISA plate was coated with dsDNA (50 ug/mL). After the synthetic peptide (RAGTDEGFG) was incubated for two hours with anti-dsDNA antibodies at RT, the reaction mixture was transferred to dsDNA coated wells. The amount of human antibodies bound to the plate was determined by using an antihuman IgG Fc specific alkaline phosphatase conjugate (Sigma). Following another washing, the substrate was added to the plate for color development.

### 4.4. Serum binding to synthetic peptides

IMMULON plates were used to detect two groups of sera; 30 SLE patients' sera and 60 control sera that include 15 rheumatoid arthritis, 15 polymyositis dermatomyositis, 15 sclerodema, 15 anti-Ro/anti-La positive sera, and 30 normal sera. The plates were coated with peptide RAGTDEGFG (100 ug/mL) and incubated at 4°C overnight. The wells were washed and blocked with blocking solution. Sera diluted 1/100 with PBS were added to the peptide-coated wells and incubated for 2 hours at room temperature. After washing, binding of IgG was detected using goat antihuman IgG Fc fragment specific alkaline phosphatase conjugate (Sigma).

## 5. RESULTS

### 5.1. Detection of anti-dsDNA antibody reaction with burkholderia fungorum bacterial cell lysates by Western Blot

**Figure 1:**
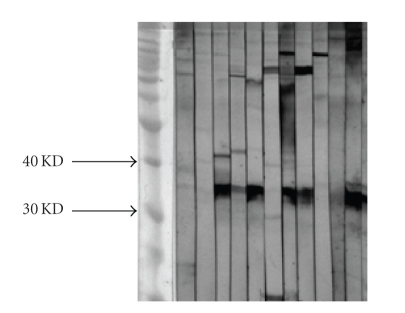
12 purified anti-dsDNA antibodies from SLE patients. Seven of these purified antibodies can bind a 35 KD burkholderia fungorum bacterial protein in Western Blot.

12 SLE patients sera with anti-dsDNA antibodies were used as a source for the affinity purification of antibodies. We purchased burkholderia fungorum bacterial cells from ATCC. We tested 12 purified anti-dsDNA antibodies from SLE patients and found that seven of these purified antibodies can bind the burkholderia fungorum bacterial protein in Western Blot (Figure 1).

### 5.2. Purified bacterial protein from burkholderia fungorum

**Figure 2:**
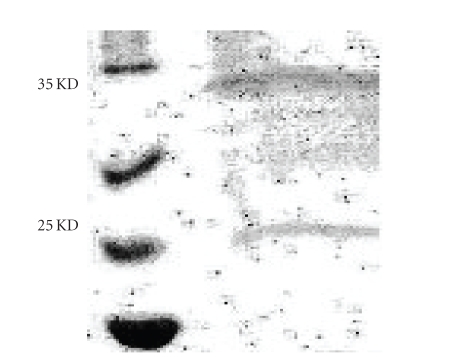
Burkholderia fungorum bacterial protein purified by anti-dsDNA antibodies affinity column. The eluate from the column was examined by 12.5% SDS gel. The result shows that purified proteins contain two major bands, one is 35 KD bacterial protein and the other one is 25 KD bacterial protein.

**Figure 3:**
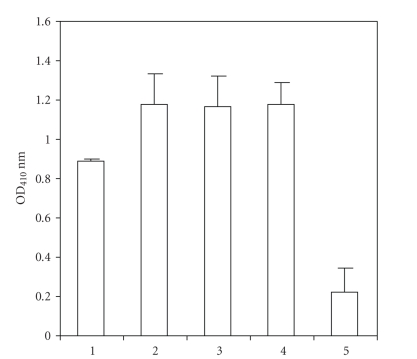
Testing 4 affinity purified human IgG anti-dsDNA antibodies in binding to the purified bacterial antigen in ELISA. ELISA plates were coated with the purified bacterial antigen (50 *μ*g/mL). Lanes 1–4 contain purified anti-dsDNA antibodies (1 *μ*g/100 *μ*L) binding to purified bacterial antigen and lane 5 has CF II as control binding to purified bacterial antigen. Anti human IgG Fc fragment specific alkaline phosphatase conjugate was used to detect anti-dsDNA antibody binding. The data were expressed as the mean ± SD.

**Figure 4:**
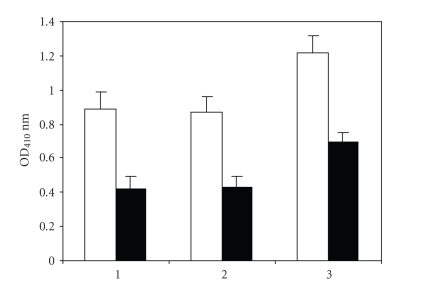
Inhibition of anti-dsDNA antibodies binding to dsDNA antigen by purified bacterial antigen. ELISA plates were coated with dsDNA (50 *μ*g/mL). Lane 1, 2, and 3 are three different patients anti-dsDNA antibodies. The light column shows the patient purified anti-dsDNA antibodies (1.25 *μ*g/100 uL) binding to dsDNA antigen, and dark column shows the patient purified anti-dsDNA antibodies (1.25 *μ*g/100 uL) mixed with purified bacterial antigen(10 *μ*g/100 uL). The data were expressed as the mean ± SD.

**Figure 5:**
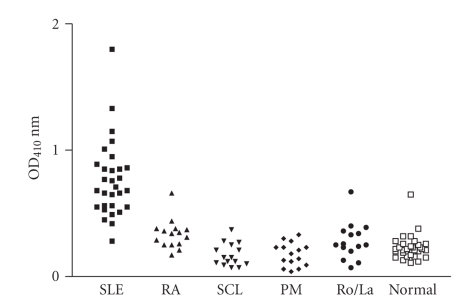
Reaction of SLE sera and control sera with the purified bacterial antigen. The sera were 1/100 in PBS and added into purified bacterial antigen-coated ELISA plates and the reaction of sera antibodies with the purified bacterial antigen were detected by goat antihuman IgG Fc fragment specific alkaline phosphatase conjugate. The 30 SLE sera and the control sera include 15 rheumatoid arthritis, 15 polymyositis dematomyositis, 15 sclerodema, 15 Ro/La positive sera, and 30 normal sera. The mean reading of 30 SLE sera bound with purified bacterial antigen was statistically different from that of control sera (*P* < .0001 by unpaired *t*-test).

We used one of the SLE patients anti-dsDNA antibodies that reacts with the burkholderia fungorum bacterial protein to make an anti-dsDNA antibody affinity column. We used this affinity column to purify the burkholderia fungorum bacterial protein. We found that purified burkholderi fungorum bacterial protein contains two major bands in 12.5% SDS gel. One is about 35 KD and another one is about 25 KD, as seen in Figure 2. We tested 4 purified anti-dsDNA antibodies for binding with the burkholderia fungorum bacterial protein by ELISA. All 4 anti-dsDNA antibodies were reactive with the burkholderia fungorum bacterial protein, and the control IgG was very weakly reactive (Figure 3). The burkholderia fungorum bacterial protein was studied for the ability to inhibit anti-dsDNA antibody activity. As seen in Figure 4, anti-dsDNA antibody preparations bound to dsDNA antigen can be inhibited by the bacterial protein. We tested the SLE sera, control sera, and normal sera. As seen in Figure 5, the burkholderia fungorum bacterial protein was reactive with SLE sera with anti-dsDNA antibodies. The reaction can be blocked by dsDNA antigen. In control sera, some rheumatoid arthritis sera with anti-ssDNA antibodies were reactive to burkholderia fungorum bacterial protein. We picked these sera and did inhibition by ssDNA and synthesized peptide. The result shows that reaction can be blocked by ssDNA antigen. The differences in means of OD_410_ reading between the SLE sera (mean = 0.7617, *N* = 30) and normal sera (mean = 0.2320, *N* = 30) when they bound to the bacterial antigen were statistically compared by *t*-test (using GraphPad prism), and the differences were very significant (*P* < .0001).

### 5.3. Sequence analysis of 25 KD and 35 KD bacterial proteins

The electrophoresed purified burkholderia fungorum proteins from an anti-dsDNA affinity column were transferred on to polyvinyl difluoride (PVDF) membranes. The bacterial proteins were screened by Western Blot using anti-dsDNA antibodies. The two immunoreactive bands (25 KD and 35 KD) were excised from the PVDF and sequenced. A protein partial sequence of RAGTDEGFG from the protein band is shared with burkholderia bacterial transcription regulator protein sequence.

### 5.4. Confirming the binding of anti-dsDNA antibodies to the peptide by chemical synthesis of peptides

**Figure 6:**
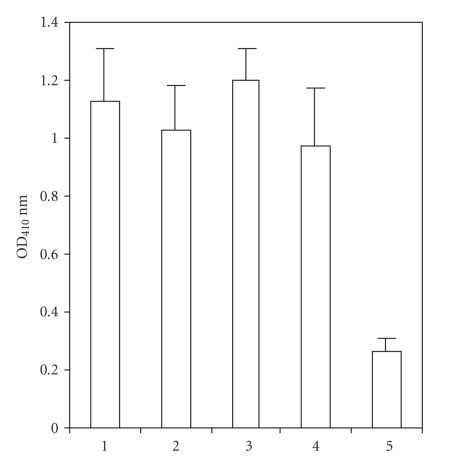
Testing 4 affinity purified human IgG anti-dsDNA antibodies in binding to the synthetic peptide in ELISA. ELISA plates were coated with synthetic peptide (100 *μ*g/mL). Lanes 1–4 contain purified anti-dsDNA antibodies (1 *μ*g/100 *μ*L) binding to synthetic peptide and lane 5 has CF II as control binding to synthetic peptide. Antihuman IgG Fc fragment specific alkaline phosphatase conjugate was used to detect anti-dsDNA antibody binding. The data were expressed as the mean ± SD.

RAGTDEGFG peptide which is shared with burkholderia bacterial transcription regulator protein sequence was synthesized. We tested 4 purified anti-dsDNA antibodies for binding with the RAGTDEGFG peptide by ELISA. All 4 anti-dsDNA antibodies were reactive with the synthesized peptide, and the control IgG was very weakly reactive (Figure 6). We also compared RAGTDEGFG peptide with scramble control peptide FGAGTRDGE. Anti-dsDNA antibodies were more strongly reactive with RAGTDEGFG peptide than scrambled control peptide FGAGTRDGE.

### 5.5. Inhibition assay

**Figure 7:**
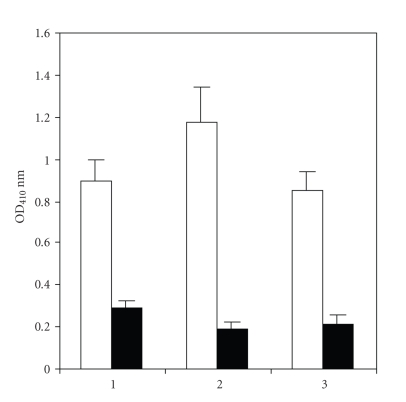
Inhibition of anti-dsDNA antibodies binding to dsDNA antigen by synthetic peptide (RAGTDEGFG). ELISA plates were coated with dsDNA (50 *μ*g/mL). Lane 1, 2, and 3 are three different patients anti-dsDNA antibodies. The light column shows the patient purified anti-dsDNA antibodies (1.25 *μ*g/100 uL) bind to dsDNA antigen, and dark column shows the patient purified anti-dsDNA antibodies (1.25 *μ*g/100 uL) mixed with synthetic peptide (3.4 *μ*g/100 uL). The data were expressed as the mean ± SD.

The synthesized peptide (RAGTDEGFG) was studied for the ability to inhibit anti-dsDNA antibody activity. As seen in Figure 7, anti-dsDNA antibody preparations bound to dsDNA antigen can be inhibited by the synthetic peptide.

### 5.6. Binding of SLE sera with synthesized peptide RAGTDEGFG in ELISA

**Figure 8:**
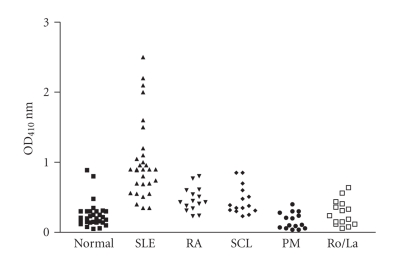
Reaction of SLE sera and control sera with the peptide. The sera were diluted 1/100 in PBS and added to peptide-coated ELISA plates and the reaction of sera antibodies with the peptide were detected by goat antihuman IgG Fc fragment specific alkaline phosphatase conjugate. The 30 SLE sera and the control sera include 14 rheumatoid arthritis, 12 polymyositis dematomyositis, 14 sclerodema, 15 Ro/La positive sera, and 30 normal sera. The mean reading of 30 SLE sera bound with peptide was statistically different from that of control sera (*P* < .0001 by unpaired *t*-test).

We tested the SLE sera, control sera, and normal sera, as seen in Figure 8. The synthetic peptide was reactive with SLE sera with anti-dsDNA antibodies. The reaction can be blocked by dsDNA antigen. In control sera, some sera with anti-ssDNA antibodies were reactive to synthesized peptide. We picked these sera and did inhibition by ssDNA and synthesized peptide. The results show that reaction can be blocked by ssDNA antigen. The differences in means of OD_410_ reading between the SLE sera (mean = 1.015, *N* = 30) and normal sera (mean = 0.2513, *N* = 30) when they bound to the synthetic peptide were statistically compared by *t*-test (using GraphPad prism), and the differences were very significant (*P* < .0001). The differences in means of OD_410_ reading between the SLE sera (mean = 1.015, *N* = 30) and control sera (mean = 0.3383, *N* = 60) when they bound to the synthetic peptide were statistically compared by *t*-test (using GraphPad prism), and the differences were very significant (*P* < .0001).

## 6. DISCUSSION

Environmental pathogens have long been suspected to be inducers of autoimmune disease. Autoimmune NZB mice raised in a germ-free environment develop reduced titers of auto-antibodies, and elevated titers of anti-dsDNA antibodies can be found in patients with microbial infections, suggesting that microbial stimulation of the immune system helps stimulate the induction of auto-antibodies in some way [11, 12]. Anti-dsDNA antibodies derived from naïve lupus prone mice can bind to the cell surfaces of murine endogenous microbial flora [13]. Monoclonal anti-dsDNA antibodies derived from mice and humans with SLE bind to the glycolipid components of the mycobacterial cell wall [14], suggesting that bacterial antigens may elicit these antibodies. This crossreactivity, known as molecular mimicry, offers a potential explanation for the development of autoimmunity.

In a previous study, we demonstrated that purified anti-dsDNA antibodies specifically bind a 15-mer peptide ASPVTARVLWKASH [3], and the 15-mer peptide partial sequence ARVLWKASH is shared with burkholderia bacterial cytochromeB 561 partial sequence ARVLWRATH. Burkholderia bacteria are rod-shaped, motile, gram-negative bacteria that includes both human and plant pathogens as well as environmentally important bacteria. The genus Burkholderia currently comprises 19 named species [8]. The clinical significance of Burkholderia cepacia, originally described as a plant pathogen in the 1950s [15], increased in the 1980s as it emerged as an opportunistic pathogen, colonizing the lungs of cystic fibrosis patients [16–18], and immunocompromised individuals [19–21]. At its peak incidence in the mid 1980s, 20% of colonized patients suffered from severe progressive respiratory failure with bacteremia which was often fatal [16, 22].

In this study, we have demonstrated that purified anti-dsDNA antibodies from lupus patients can react with burkholderia fungorum bacterial cell lysates in Western blot. We used one of the SLE patients anti-dsDNA antibodies that reacts with the burkholderia fungorum bacterial protein to make an anti-dsDNA antibodies affinity column. We used this affinity column to purify the burkholderia fungorum bacterial protein. We found that purified burkholderi fungorum bacterial protein contains two major bands in 12.5% SDS gel, one is about 35 KD and the other one is about 25 KD. We tested purified human anti-dsDNA antibodies binding to purified bacterial antigen compared with normal IgG binding. We also used purified bacterial antigen to block the anti-dsDNA antibodies binding to dsDNA antigen. The results show that anti-dsDNA antibody binding to purified bacterial antigen is specific. We tested 3 groups of sera for binding studies with the purified bacterial antigen. The results show that sera with anti-dsDNA antibodies bind to purified bacterial antigen more strongly than other control groups. We obtained protein partial sequence of RAGTDEGFG which is shared with burkholderia bacterial transcription regulator protein sequence. We tested 3 groups of sera for binding studies with the RAGTDEGFG peptide. The results show that sera with anti-dsDNA antibodies bind to RAGTDEGFG peptide and are higher than other control groups. In a previous study, we used a patient anti-dsDNA antibody to select phage clones and four positive clones were found [3]. Those four clones sequences are clone B (ASPVTARVLWKASHV), clone C (VSSLVLLSHGGPHSS), clone D (IMVLCPLWLGTTS), and clone E (AVAHVTSRRVPRWSAA). The clone B partial sequence ARVLWKASH is shared with burkholderia bacterial cytochromeB 561 partial sequence. The clone C partial sequence LVLLSHGGPH is shared with burkholderia bacterial transcriptional regulator partial sequence. We used the same patient's anti-dsDNA antibody to make an antibody affinity column for purified burkholderia bacterial protein. We obtained protein partial sequence of RAGTDEGFG which is shared with burkholderia bacterial transcription regulator protein sequence, but we did not obtain burkholderia bacterial cytochrome B561 protein sequence from purified bacterial antigen. It is possible that the anti-dsDNA antibody affinity column can not efficiently isolate enough burkholderia bacterial cytochrome B561 protein for sequencing. We also compared burkholderia bacterial cytochrome B561 protein (186 amino acids) with burkholderia bacterial transcription regulator (308 amino acids) protein sequence. The two proteins sequences share 29 amino acids and 20 amino acid positions have the same charge. These data support our hypothesis that the origin of anti-dsDNA antibodies in SLE may be associated with burkholderia bacterial infection. The burkholderia bacterial infection is an environmental risk factor that may be most closely associated with SLE. The development of auto-antibodies is a common feature in autoimmune diseases. Interactions between auto-antigens and auto-antibodies have to date mainly been analyzed by truncated subclones or synthetic peptides mimicking the proposed target epitopes. However, recent findings and reviews have implied that most autoepitopes may be conformation-dependent [23], thus stressing the need to develop procedures to analyze in detail which functional domain of the auto-antigen could be immunodominant. Immunization of non-autoimmune mice with Burkholderia antigens and its selected peptides would support our hypothesis if autoimmunity is induced.

Clearly, other environmental factors such as Epstein-barr virus infection [24, 25] as well as genetic and immunologic factors may also be important. Continued efforts in this sphere include the possible development of an animal model that could provide fruitful areas of investigation.
